# Implant Preference and Clinical Outcomes of Patients with Staged Bilateral Total Knee Arthroplasty: All-Polyethylene and Contralateral Metal-Backed Tibial Components

**DOI:** 10.3390/jcm12237438

**Published:** 2023-11-30

**Authors:** Luboš Nachtnebl, Vasileios Apostolopoulos, Michal Mahdal, Lukáš Pazourek, Pavel Brančík, Tomáš Valoušek, Petr Boháč, Tomáš Tomáš

**Affiliations:** 1 First Department of Orthopaedic Surgery, St. Anne’s University Hospital, 602 00 Brno, Czech Republic; lubos.nachtnebl@fnusa.cz (L.N.); vasileios.apostolopoulos@fnusa.cz (V.A.); michal.mahdal@fnusa.cz (M.M.); lukas.pazourek@fnusa.cz (L.P.); pavel.brancik@fnusa.cz (P.B.); tomas.valousek@fnusa.cz (T.V.); 2 Faculty of Medicine, Masaryk University, 625 00 Brno, Czech Republic; 3 Institute of Solid Mechanics, Mechatronics and Biomechanics, Faculty of Mechanical Engineering, Brno University of Technology, 601 90 Brno, Czech Republic; 201394@vutbr.cz

**Keywords:** bilateral knee replacement, total knee arthroplasty, all-polyethylene tibia, metal-backed tibia, staged bilateral knee arthroplasty

## Abstract

Numerous studies have compared metal-backed components (MBTs) and all-polyethylene tibial components (APTs), but none of them specifically analysed the clinical results and the overall patient preference in patients who had undergone a staged bilateral knee replacement. The purpose of this study is to compare clinical results, perceived range of motion, and overall implant preference among patients who had undergone staged bilateral knee replacement with an APT and contralateral knee replacement with MBTs. A dataset of 62 patients from a single centre who underwent staged bilateral TKA between 2009 and 2022 was selected and retrospectively analysed. Tibial component removal was performed in three knees overall, all of which had MBTs. The mean measured Knee Score (KS) of knees with APTs was 78.37 and that of contralateral knees with MBTs was 77.4. The mean measured Function (FS) of knees with APTs was 78.22, and that of contralateral knees with MBs was 76.29. The mean flexion angle of knees with APTs was 103.8 and that for knees with MBTs was 101.04 degrees. A total of 54.8% of the patients preferred the knee that received APTs over contralateral MBTs. In our cohort, TKA with an APT in one knee and an MBT in the contralateral knee recorded similar clinical results and perceived ranges of motion. Patients in general preferred the knee that received an APT over contralateral knee with an MBT.

## 1. Introduction

Total knee arthroplasty (TKA) is one of the most frequent orthopaedic procedures and the definitive treatment of knee arthritis. Even if promising new technologies have been developed in knee arthroplasty, implant selection is still discussed [1,2]. Most total knee replacements have been performed with metal-backed tibial components (MBTs). All-polyethylene tibial components (APTs) are primarily implanted in older and low-demand patients [3]. Numerous studies have compared MBTs and APTs, describing similar clinical outcomes and implant survivorship [4,5,6]. The first-generation APT designs often failed to aseptic loosening; thus, many orthopaedic surgeons are still reluctant to use APTs [7]. Nowadays, considering improvements in implant design, material quality, and the economic strain on health care, APT utility is regaining popularity [8,9].

Our institution has a long tradition in arthroplasty with APTs, choosing APTs not only in cases of elder patients. A previous study described excellent long-term clinical results and survivorship of APTs [10]. Moreover, in a clinical comparison of 812 patients with NexGen TKA APTs and MBTs, we suggest that APTs are equal or even superior to metal-backed components across the age categories [3]. Further examination of the topic in a biomechanical analysis on APTs showed a similar induced response in patients of the 60–70-year-old age groups as well as remodelling and modelling of the periprosthetic tibia, which is a beneficial factor in implant survivorship [11].

Despite numerous studies that have compared APTs with MBTs, none of them specifically analysed the clinical outcomes and the overall patient preference in patients who had undergone a staged bilateral knee replacement, having one knee with MBTs and the contralateral knee with APTs, implanted in a single orthopaedic department using the same surgical technique. The purpose of this retrospective study was to compare clinical results, perceived range of motion, and overall preference among patients who had undergone staged bilateral TKA with APTs and contralateral TKA with MBTs.

## 2. Materials and Methods

### 2.1. Sample Characteristics

**Figure 1:**
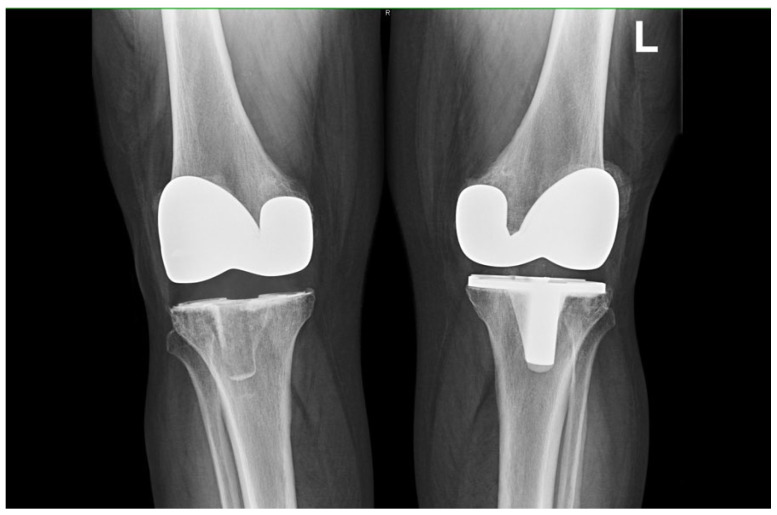
Radiological imaging of representative patient with staged bilateral TKA, right side with TKA APT implanted and left side with MBT.

For this retrospective comparative study, a dataset of 62 patients from a single centre who underwent staged bilateral TKA between 2009 and 2022 was selected. All of the patients had one knee with an MBT and an APT in the contralateral knee (Figure 1). In total, this represented 124 knee replacements, 34 of which had an APT on the right side, 28 with an APT on the left side, 34 with an MBT on the left side, and 28 with an MBT on the right side. There were 37 females and 25 males. The only indication for TKA was primary knee arthritis; patients with post-traumatic or other non-primary knee arthritis were excluded from this study (Table 1).

**Figure 2:**
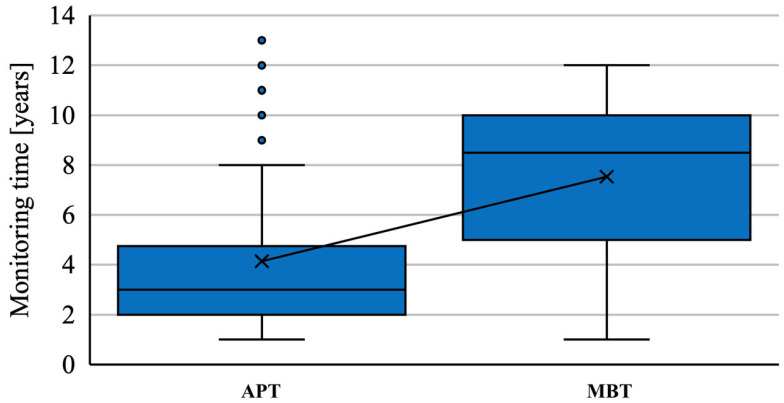
Follow-up patients with staged bilateral TKA in our institution from 2009 to 2022 for each implant type.

Patients were followed up for at least 1 year, from 1 to 12 years postoperatively. The mean follow-up was 4.14 years for APTs and 7.45 years for MBTs (Figure 2). The mean age at the time of MBT implantation was 69.9 years and that at the time of APT implantation was 74.1 years. Exclusion criteria were a minimum 1-year follow-up and a maximum interval of 10 years between the first TKA and the TKA of the other side.

### 2.2. Evaluation

The primary efficacy endpoint compared was the clinical outcome of the implants as per the Knee Society Score before and after the procedure, which was assessed at a minimum of 1-year follow-up and eventually modified at the last examination, with both the knee-specific score (KS) and general functional score (FS) [12]. Both scores range from 0 to 100 (excellent) [12]. The secondary efficacy endpoint was the determination of the maximum range of motion in patients observed at a minimum of 1-year follow-up and eventually modified at the last examination. The range of motion was measured with a goniometer (universal goniometer) while the patient was lying on the exam table. The last efficacy point was an examination of the subjective implant preference of the patients between APTs and MBTs. Patients were asked directly to choose the knee they were satisfied with the most.

### 2.3. Implant Types

**Figure 3:**
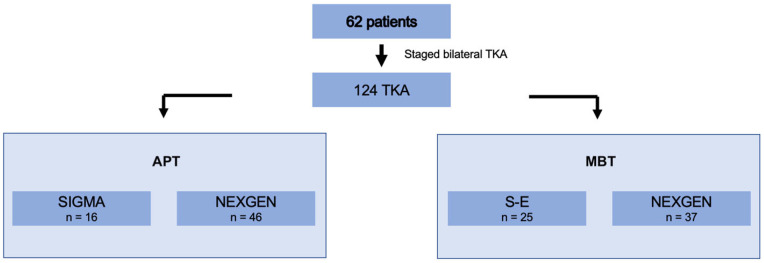
Sample distribution and TKA system variants used in staged bilateral TKA in our institution from 2009 to 2022.

Four different systems of cemented TKA CR, two per type, were used (Figure 3). The all-polyethylene knee system variants included Sigma DePuy (*n* = 16) and NexGen Zimmer (*n* = 46). The metal-backed knee system variants included Search Evolution Aesculap (*n* = 37) and NexGen Zimmer (*n* = 25). The implant selection was based on current research evidence and advances in the field of orthopaedics to ensure the best outcomes for each patient.

### 2.4. Surgical Technique

In all cases, the same surgical technique was utilised, following the standard principles used in our institution. The mechanical alignment concept and medial parapatellar approach to the knee were employed using a tourniquet cuff inflated at 300 mmHg. Patellar resurfacing was performed in select cases, while patellar denervation with electrocautery and osteophyte removal were conducted in all cases. Bone cement was applied to the implants, with a small amount pressed into the cancellous bone. At the end of the procedure, two suction drains were inserted to capture the blood loss during the first 24 h, and 1 g of tranexamic acid was applied intra-articularly. All patients received a single preoperative dose of prophylactic antibiotics (cefazolin) before the inflation of the tourniquet and 3 doses postoperatively. All patients participated in the same postoperative course and physical therapy during their 7-day hospital stay.

### 2.5. Statistical Methods

The statistical analysis was carried out using R version 4.0.5 (Bell Laboratories, Murray Hill, NJ, USA) software. The Fischer exact test was conducted to compare categorical variables between the two groups. A significance level of *p* < 0.05 was used. A 95% confidence interval (95% CI) was calculated. The non-parametric Mann–Whitney test was used to compare the clinical outcomes between the implants.

## 3. Results

Tibial component removal was performed in three knees overall, all of which had an implanted MBT, and there was no statistically significant relationship between the survival rate and the type of implant (*p* = 0.2439). The cause of removal in two of the cases was due to aseptic loosening of the tibial component. The first occurred 4 years after the implantation and the second 10 years after the implantation. The last case of tibial component removal was because of arthrofibrosis, a reduced range of motion to 60 degrees of flexion, and an extension deficit of 5 degrees.

### 3.1. Functional Outcome

**Figure 4:**
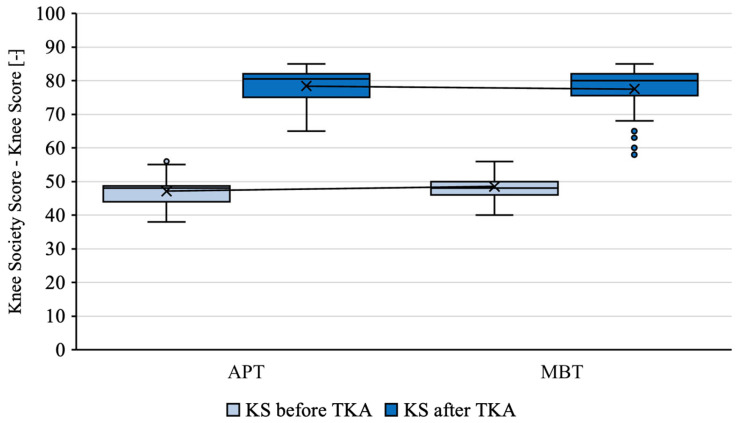
KS of implants in patients with staged bilateral TKA in our institution from 2009 to 2022.

**Figure 5:**
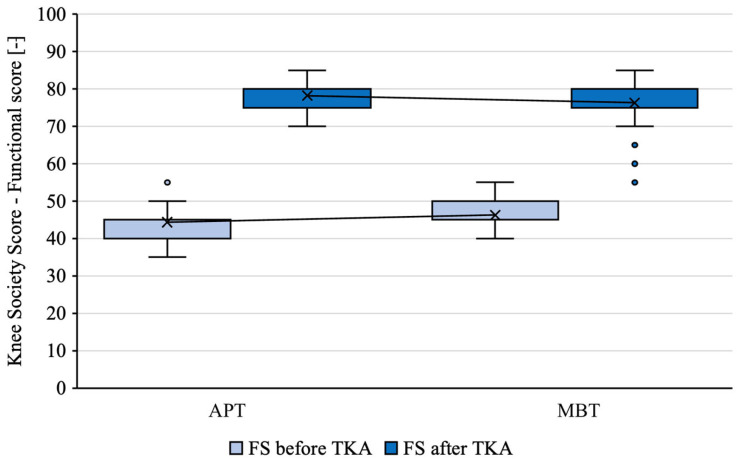
FS of implants in patients with staged bilateral TKA in our institution from 2009 to 2022.

The mean measured KS of knees before the TKA with an APT was 47.11 and the mean KS of contralateral knees before the TKA with an MBT was 48.54 (*p* = 0.01684) (Figure 4). The mean measured FS of knees before the TKA with an APT was 44.35 and the mean FS of contralateral knees before the TKA with an MBT was 46.29 (*p* = 0.01352) (Figure 5). See Table 2.

The mean measured KS of knees with an APT was 78.37 and the mean KS of contralateral knees with an MBT was 77.4 (*p* = 0.29372) (Figure 4). The mean measured FS of knees with an APT was 78.22 and the mean FS of contralateral knees with an MBT was 76.29 (*p* = 0.72786) (Figure 5). See Table 3.

For both of the tibial components, there was a statistically significant improvement in FS and KS after the TKA (*p* < 0.00001).

### 3.2. Range of Motion

**Figure 6:**
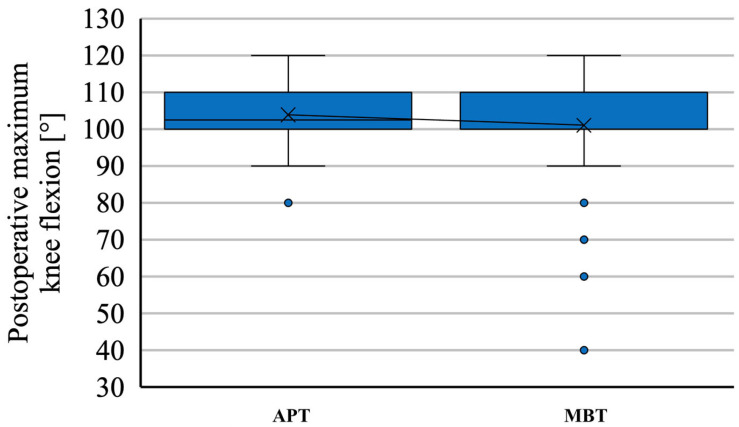
Maximum knee flexion in patients with staged bilateral TKA in our institution from 2009 to 2022.

The mean knee flexion angle of knees with an APT was 103.8 (±8.21) degrees, and the mean flexion contracture was 6.25 degrees and occurred in eight knees. On the contrary, the mean knee flexion angle of contralateral knees with an MBT was 101.04 degrees (±14.29) (*p* = 0.52218), and the mean flexion contracture was 8 degrees and occurred only in five knees (*p* = 0.5593) (Figure 6). Manipulation under anaesthesia was necessary in one case because of stiffness in the APT. No cases were treated with arthroscopic or open lysis of adhesions.

### 3.3. Implant Preference

**Figure 7:**
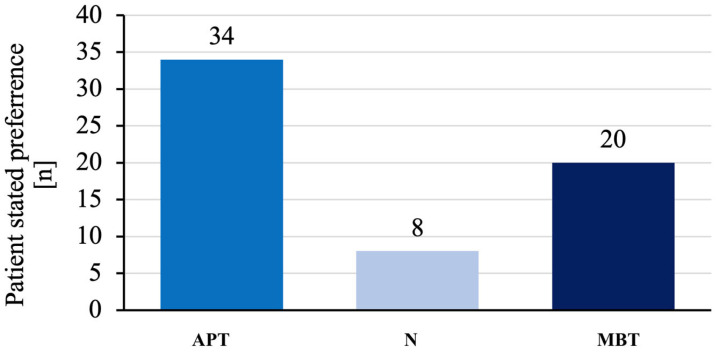
Patient-stated implant preference in staged bilateral TKA in our institution from 2009 to 2022.

At the follow-up, patients were asked to directly compare their knees. A total of 54.8% of the patients preferred the knee that received an APT over the contralateral MBT. Meanwhile, 12.9% of the patients found no difference and 32.3% preferred the MBT (Figure 7).

## 4. Discussion

In modern orthopaedics, modular MBTs are preferred by the majority of orthopaedic surgeons over monoblock APTs [13]. The poor clinical results described in the 1980s notably lowered the utilisation rates of APTs [14]. Even though the design of these components and the polyethylene material used to make them have been significantly improved, APTs are still primarily recommended for older and low-demand patients [15]. Despite numerous papers comparing APTs and MBTs, there is no study specifically comparing these tibial components in bilateral TKA. This study compared the clinical outcomes of an APT in one knee and an MBT in the contralateral knee of the same patient in staged bilateral TKA.

Previous studies comparing these implants presented similar or even superior mid-term clinical survivorship of APTs [16,17]. In this study, out of a total of 124 TKAs, removal of the tibial component was necessary in three cases, and all of them were MBTs; removal of APTs was not necessary. There were no cases of polyethylene insert exchange, whereas modularity is considered to be a major advantage of MBTs [18]. Nouta et al., in meta-analysis comprising more than 12,500 TKAs, found no differences in clinical and functional outcomes between the two implants. The mean KS and FS for APTs and MBTs were found to be similar [19]. Another recent study comparing the two tibial components observed similar clinical results using the same evaluation method [20]. In the literature, there are only a few specific clinical comparisons between the components in bilateral staged TKA [21,22]. Our study recorded comparable clinical and functional outcomes using the Knee Society Score. The mean KS of APTs at the last follow-up (78.37) was similar to the mean KS of contralateral knees with MBTs (77.4). Analogously, the mean FS of APTs at the last follow-up (78.22) was comparable with the mean FS of contralateral knees with an MBT (76.29).

A recent study comparing the medium-term outcomes of the two implants described a similar range of motion in patients older than 70 years of age [20]. In this study, we recorded a similar maximal knee flexion when comparing the two tibial components. A slightly greater mean maximal flexion was measured in knees with APTs (103.8 degrees); on the contrary, the mean maximal flexion of knees with MBTs was 101.04 degrees. The difference in maximal knee flexion was not found to be significant. Flexion contracture was recorded in eight knees with APTs and five knees with MBTs. There was only a single patient with an APT undergoing manipulation under anaesthesia for stiffness.

Since APTs are primarily recommended for elderly patients, the implantation of the APTs was, in most cases, performed years after the implantation of MBTs. Additionally, we found excellent results of APTs in older patients [10]. Consequently, even if these older patients have previously received an MBT on the contralateral side during a previous surgery at a younger age, they still receive an APT. This explains the significant difference in the age at inclusion of the patients as well as the longer follow-up of patients with MBTs. Minimising the variation in follow-up durations was not feasible as we compared results for the same patient, and APTs were mostly implanted years later. Another issue we need to highlight is the lower KS and FS at the implantation of APTs. This could be probably explained by the age at implantation as the scoring system is associated with patient infirmity, especially in the case of the FS.

One crucial aspect following a TKA implantation is the overall satisfaction of the patient [23]. Therefore, we took into account the subjective preference of each patient by directly asking them to choose between the APT and the contralateral MBT. A similar method was used in a study comparing the overall preference among patients who had undergone staged bilateral TKA with a customised implant in one knee and an off-the-shelf implant in the contralateral knee [24]. In our query, 54.8% of the patients preferred the knee that received the APT over that with the contralateral MBT. Only 12.9% of the patients could not decide which of the knees they favoured.

The present study has several strengths that contribute to its reliability. Firstly, it possesses an appropriate sample size consisting of patients who underwent bilateral TKA. Additionally, the surgical technique employed was consistent across all patients, and the mean follow-up duration is notable. These strengths enabled us to effectively compare the performance of the two tibial component types. However, it is important to acknowledge the limitations of our study. Firstly, it had a retrospective design and varying follow-up durations for the studied knees. Additionally, some of the TKA cases only had a 1-year follow-up. Secondly, the sample consisted of four different systems of cemented TKA, with two variants for each type, which introduced some degree of variability. Considering these limitations, we believe that the obtained results are still meaningful and provide valuable insights into the comparison of the two tibial component types.

## 5. Conclusions

The present study provides evidence that TKA with an APT in one knee and an MBT in the contralateral knee exhibits comparable clinical and functional outcomes. Patients generally expressed a preference for the knee that received the APT over that with the contralateral MBT. Taking into account their limitations, our findings could influence implant selection, encouraging more frequent utilisation of TKAs with an APT.

## Figures and Tables

**Table 1:** Sample characteristics.

Features	Bilateral TKA
Number of patients (knees)	62 (124)
Age at inclusion (years)	
APT	74.1 ± 3.56 (66–82)
MBT	69.9 ± 3.69 (61–79) *p* < 0.00001
Sex	
Female	37 (59.7%)
Male	25 (40.3%)
Average follow-up (years)	
APT	4.14 ± 3.19 (1–12)
MBT	7.45 ± 3.01 (1–12) *p* < 0.00001
Diagnosis	
Primary osteoarthritis	124 (100%)
Surgical approach	
Medial parapatellar	124 (100%)

**Table 2:** Preoperative KS and FS of patients with staged bilateral TKA in our institution from 2009 to 2022.

Overall	KS: APT	KS: MBT	FS: APT	FS: MBT
Mean	47.11	48.54	44.35	46.29
Std. Dev	4.00	3.56	4.09	3.83
Median	48	48	45	45
N.Valid	62	62	62	62

**Table 3:** Postoperative KS and FS at the last follow-up of patients with staged bilateral TKA in our institution from 2009 to 2022.

Overall	KS: APT	KS: MBT	FS: APT	FS: MBT
Mean	78.37	77.4	78.22	76.29
Std. Dev	5.11	6.87	4.44	7.06
Median	80.5	80	80	80
N.Valid	62	62	62	62

## Data Availability

The data presented in this study are available on request from the corresponding author. The data are not publicly available for privacy reasons.
